# Relative increase of memory B-cell subsets under s.c. B-cell-depleting therapies in multiple sclerosis

**DOI:** 10.3389/fimmu.2026.1812392

**Published:** 2026-06-15

**Authors:** Adriana Krenz, Anna-Lena Krickl, Felix Burner, David Freudenstein, Constantin Träger, Timo Wirth, Luisa Klotz, Klemens Angstwurm, De-Hyung Lee, Ralf A. Linker, Stefanie Haase

**Affiliations:** 1 Department of Neurology, University Hospital Regensburg, Regensburg, Germany; 2 Department of Neurology, University Hospital Münster, University of Münster, Münster, Germany

**Keywords:** B cell depletion, immunotherapy, memory B cells, monoclonal antibodies, multiple sclerosis

## Abstract

**Background:**

Monoclonal antibodies targeting CD20-expressing B cells constitute effective therapies for people with multiple sclerosis (pwMS) and can be administered intravenously (i.v.) or subcutaneously (s.c.). However, little is known about the differences in immunomodulatory effects of distinct B-cell depletion therapies.

**Methods:**

We performed detailed characterizations of the immune cell composition in blood samples of pwMS treated with either i.v. [ocrelizumab (Ocre); *n* = 25] or s.c. [ofatumumab (Ofa); *n* = 25 or Ocre *n* = 25] administered anti-CD20 therapy compared to healthy controls (HC, *n* = 20). In addition, blood samples from *n* = 25 pwMS who switched their anti-CD20 therapy from i.v. Ocre to s.c. Ofa injection were analyzed before therapy change and after 4 and 8 weeks. We performed flow cytometry analysis of whole blood samples and purified peripheral blood mononuclear cells for intracellular cytokine staining.

**Results:**

Both anti-CD20 mAbs resulted in a depletion of CD19^+^ B cells [median (IQR) HC: 16.5 (10.1–22.2), Ocre i.v.: 0.4 (0.1–0.9), Ocre s.c.: 0.8 (0.4–1.2), Ofa s.c.: 1.7 (0.8–2.2)] and CD20^+^ T cells [median (IQR) HC: 9.1 (6.6–16), Ocre i.v.: 0.6 (0.1–1), Ocre s.c.: 0.3 (0.2–0.5), Ofa s.c.: 0.1 (0.1–0.4)] in peripheral blood. More in-depth characterization of the patients’ remaining B cells indicated higher percentages of memory B cells in Ocre and Ofa s.c. compared to Ocre i.v. treated pwMS [median (IQR) Ocre i.v.: 6.6 (0–13), Ocre s.c.: 18.4 (4–26.8), Ofa s.c.: 26.4 (21.1–30)]. In pwMS who switched from i.v. Ocre to s.c. Ofa therapy, the percentage of memory B cells increased over time [median (IQR) baseline: 14.6 (6.4–20.8), 4 weeks after switch: 12.1 (6–23.4), 8 weeks after switch: 22.8 (16.7–29.5)]. I.v. Ocre treatment led to significantly lower serum IgG levels in pwMS compared to HC.

**Discussion:**

Our data add to the knowledge on distinct antibody-specific properties and differential effects of s.c. versus i.v. administered B-cell depletion in pwMS. We identified a higher percentage of memory B cells in s.c. Ocre and Ofa B cell-depleted pwMS and pwMS who switched their anti-CD20 therapy from i.v. Ocre to s.c. Ofa injection. Further analysis will be needed to investigate how these observations link to safety and efficacy profiles of the different anti-CD20 therapies.

## 1 Introduction

Multiple sclerosis (MS) is an immune-mediated disease of the central nervous system (CNS) characterized by inflammation and demyelination (1, 2). For many years, MS was considered a primarily T cell-mediated disease; however, accumulating evidence has highlighted the essential role of B cells in MS immunopathogenesis.

In recent years, the development of anti-CD20 monoclonal antibodies (mAbs) has fundamentally advanced the therapeutic landscape of MS by selectively depleting CD20^+^ B and T cells, thereby suppressing disease activity and mitigating disability progression in affected individuals (3). At present, four primary anti-CD20 mAbs are utilized in the treatment of relapsing–remitting multiple sclerosis (RRMS): rituximab, ocrelizumab (Ocre), ofatumumab (Ofa), and ublituximab (4–7).

Among these, intravenous (i.v.) administration of Ocre and subcutaneous (s.c.) administration of Ofa represent two clinically approved approaches targeting the CD20 antigen on B cells via different application routes. Ocre is a humanized mAb recognizing the large extracellular loop of the CD20 antigen and is administered i.v. every 6 months at doses of 600 mg (8). Since 2024, Ocre can also be administered s.c. twice a year at a dose of 920 mg (9). Unlike Ocre, Ofa can bind two distinct regions within the large and small extracellular loops and can be self-administered by patients s.c. at monthly doses of 20 mg (6, 10, 11). While both agents demonstrate robust efficacy in reducing disease activity, the immunological consequences of these differing routes of administration remain incompletely understood (6, 7). Understanding the pharmacological and immunological differences can be critical to optimize individualized treatment strategies in MS; however, comparative data remain limited. This study investigates immunological effects in people with multiple sclerosis (pwMS) treated with either i.v. or s.c. Ocre or s.c. Ofa to further understand the consequences of different B-cell depletion therapies.

## 2 Methods

### 2.1 Standard protocol approvals, registrations, and patient consents

This study was approved by the appropriate institutional review board at the University of Regensburg according to the principles of the Declaration of Helsinki (approval number: 21-2307-101). All patients provided written informed consent.

### 2.2 Study participants

We analyzed blood samples of pwMS who provided specimens between October 2022 and August 2025 at the Department of Neurology, University Hospital Regensburg. For cross-sectional analysis, we included *n* = 25 i.v. Ocre-treated pwMS, *n* = 25 s.c. Ocre-treated pwMS, and *n* = 25 s.c. Ofa-treated pwMS. Baseline characteristics of study participants are listed in **Table 1**. All included patients were diagnosed with active MS according to the 2017 McDonald criteria and were evaluated using the Expanded Disability Status Scale (EDSS). Treatment decisions were made independently of this study by the treating neurologists. Patients were eligible if they were at least 18 years old and did not match any of the exclusion criteria: chronic kidney failure, ongoing oncologic disease, severe gastrointestinal conditions, acute or chronic infection, current post-surgical rehabilitation, and alcohol or drug abuse. To reduce potential bias, we consecutively enrolled B cell-depleted pwMS during the recruitment period and applied standardized protocols to minimize information bias. Healthy controls (HC; *n* = 20) were recruited at the University of Regensburg. *A priori* sample size calculations for the cross-sectional study (using G*Power 3.1; ANOVA, one-way, α-error 0.05, intended minimum test power 0.8) revealed a total sample size of *n* = 76 (*n* = 19 per group) to identify potential large effects (*f* = 0.4). *Post-hoc* analysis (*f* = 0.58, α-error=0.05) revealed an achieved test power of 0.99. The same procedure was conducted for longitudinal analysis of switcher; *a priori* sample size calculation (using G*Power 3.1, ANOVA, repeated measurements, within factors, α-error 0.05, intended minimum test power 0.8, *k* = 3, ε = 1) calculated a total sample size of *n* = 12 to identify large effects (*f* = 0.4). *Post-hoc* analysis (*f* = 0.46, α-error=0.05) identified an achieved test power of 0.99.

**Table 1:** Baseline characteristic of study participants.

Parameters	HC	Ocre i.v.	Ocre s.c.	Ofa s.c.	Switcher
Study participants [*n*]	20	25	25	25	25
Female/Male [*n*/*n*]	10/10	16/9	16/9	16/9	16/9
Age [median years (IQR)]	30.5 (24–52.3)	50 (39–57)	38 (26–58)	41 (31–53)	48 (32–56)
EDSS [median (IQR)]	–	4 (2–5.5)	3 (2–4)	2.5 (2–4)	3 (2.9–4.6)
Disease duration [median years (IQR)]	-	8 (3.8–19.3)	4 (2–9.3)	2.8 (1.3–15)	8.3 (4.5–16.8)
Therapy duration [median years (IQR)]	–	2.4 (1.5–3.2)	0.5 (0.5–1)	0.7 (0.08–1.25)	–

### 2.3 Blood sample acquisition and handling

Peripheral venous blood samples were drawn and transported at room temperature (RT) for further processing. Routine clinical markers were analyzed by the central laboratory at the medbo Bezirksklinikum Regensburg. Serum was extracted after centrifugation from whole blood in a Serum Gel CAT Monovette (Sarstedt) and frozen immediately at −80°C. Whole blood samples were stored in S-Monovettes EDTA KE (Sarstedt) on a rolling mixer at RT until the isolation of peripheral blood mononuclear cells (PBMCs) within 24 h.

### 2.4 Whole blood flow cytometric analysis

Fc-receptor blocking reagent (Miltenyi Biotec, #130-059-901) was added to whole blood to prevent non-specific binding of antibodies. For cell surface staining, an antibody-cocktail containing fluorochrome-labeled antibodies or the respective isotype controls as listed in **Supplementary Table 1** was incubated with 100 µL of whole blood for 10 min at 4 °C. 1:10 diluted FACS Lysing Solution (BD Bioscience, #349202) was added and incubated for 10 min at RT to lyse erythrocytes. Cells were washed with FACS buffer (1× PBS, 2 mM ETDA). Flow cytometric analyses were performed using the FACS CantoTM II (BD Bioscience) flow cytometer. Manual gating was performed using FlowJo, and a respective gating strategy is shown in **Supplementary Figure 1**. The compensation procedure is described in **Supplementary Material** (1.3 Flow cytometry).

### 2.5 Isolation of peripheral blood mononuclear cells

PBMCs were isolated via density gradient centrifugation using Pancoll (PAN Biotech, #P04-601000). The cell number was determined after Acridine Orange Propidium Iodid staining (Logos Biosystems, #F23001). A total of 1 × 10^6^ isolated PBMCs were used for intracellular staining or 4 × 10^6^ PBMCs lysed in 350 µL of TRK buffer (peqGold Total RNA Kit, VWR) and stored at −80 °C for further gene expression analysis.

### 2.6 Intracellular cytokine staining

A total of 1 × 10^6^ isolated PBMCs were stimulated using Ionomycin (1 mg/mL, Sigma-Aldrich, #I3909-1ML), Phorbol-12-myristat-13-acetat (PMA, 0.1 mg/mL, Sigma-Aldrich, #P1585-1MG), and Monensin (2 mM, BioLegend, #420701) in TexMACS Medium (Miltenyi Biotec, #130-097-196) for 3 h, 37 °C, 5% CO_2_. Dead cells were excluded by viability staining using Fixable Viability Dye eFluor 780 (eBioscience, #65-0865-14). Fc-receptor blocking reagent (Miltenyi Biotec, #130-059-901) was added to whole blood to prevent non-specific binding of antibodies. Cell surface staining was performed by staining the cells with the respective antibodies diluted in PBS listed in **Table 1** for 10 min, 4 °C. Cells were further fixed and lysed using the Foxp3/Transcription Factor Staining Buffer Set (Invitrogen, #00-5523-00) according to the manufacturer’s instructions. For intracellular staining, the respective antibodies listed in **Table 1** were used and incubated with the cells for 40 min at RT. Flow cytometric analyses were performed using the FACS CantoTM II (BD Bioscience) flow cytometer.

### 2.7 High-dimensional data analysis of flow cytometry data

For high-dimensional data analysis, the platform OMIQ was used. After compensation and quality control, data were uploaded to OMIQ and processed using the following workflow: Pregating was performed as follows: Cells of WB were gated for lymphocytes, and doublets and CD19^+^ cells were identified. Uniform Manifold Approximation and Projection (UMAP) was generated based on the expression of the markers CD19, CD20, CD27, and CD38. UMAP was computed with Euclidean distance and default parameters (15 nearest neighbors; minimum distance, 0.4). FlowSOM clustering (*k* = 4) was applied to identify four B-cell metaclusters within the UMAP-defined B-cell compartment yielding a clustering stability of 56%.

### 2.8 Statistical analysis

Statistical analysis was performed using the software GraphPad Prism 10. Four independent groups were analyzed by the non-parametric Kruskal–Wallis test and the *post-hoc* Dunn’s multiple comparisons test. All pairwise comparisons were adjusted by applying the option “correct for multiple comparisons using a statistical hypothesis testing”. Adjusted *p*-values are reported for all significant comparisons. The non-parametric Friedman test was used to determine the significance of differences of repeated measures on the same group of patients followed by the *post-hoc* Dunn’s multiple comparisons test. Data are presented as median and interquartile range (IQR). Investigators were blinded to group allocation during data collection and analyses to minimize any potential bias.

## 3 Results

### 3.1 Study participants’ characteristics

Within this study, we performed a cross-sectional comparison to investigate potential immunomodulatory differences of distinct antibodies of anti-CD20 therapies in pwMS. Here, we investigated blood samples from *n* = 20 HC, *n* = 25 pwMS treated with Ocre i.v., *n* = 25 pwMS who received Ocre s.c., and *n* = 25 pwMS who received Ofa via s.c. self-injection (Ofa). Additionally, a longitudinal study design was used to investigate pwMS who switched from i.v. Ocre to s.c. Ofa B-cell depletion (Switcher; *n* = 25) (**Table 1**). The median disease duration at the time of analysis was 8 years (IQR 3.8–19.3) in the i.v. Ocre-treated pwMS, 4 years (2–9.3) in s.c. Ocre-treated pwMS, 2.8 (1.3–15) in the Ofa group, and 8.3 years (4.5–16.8) in the Switcher group. The median EDSS score was comparable between Ocre-treated pwMS and the Switcher cohort (Ocre i.v.: 4 versus Ocre s.c.: 3 versus Switcher: 3) and lower in pwMS receiving Ofa treatment (Ofa: 2.5) but without statistically significant difference.

### 3.2 Higher percentages of memory and naïve B cells after subcutaneous Ocre and Ofa treatment

**Figure 1:**
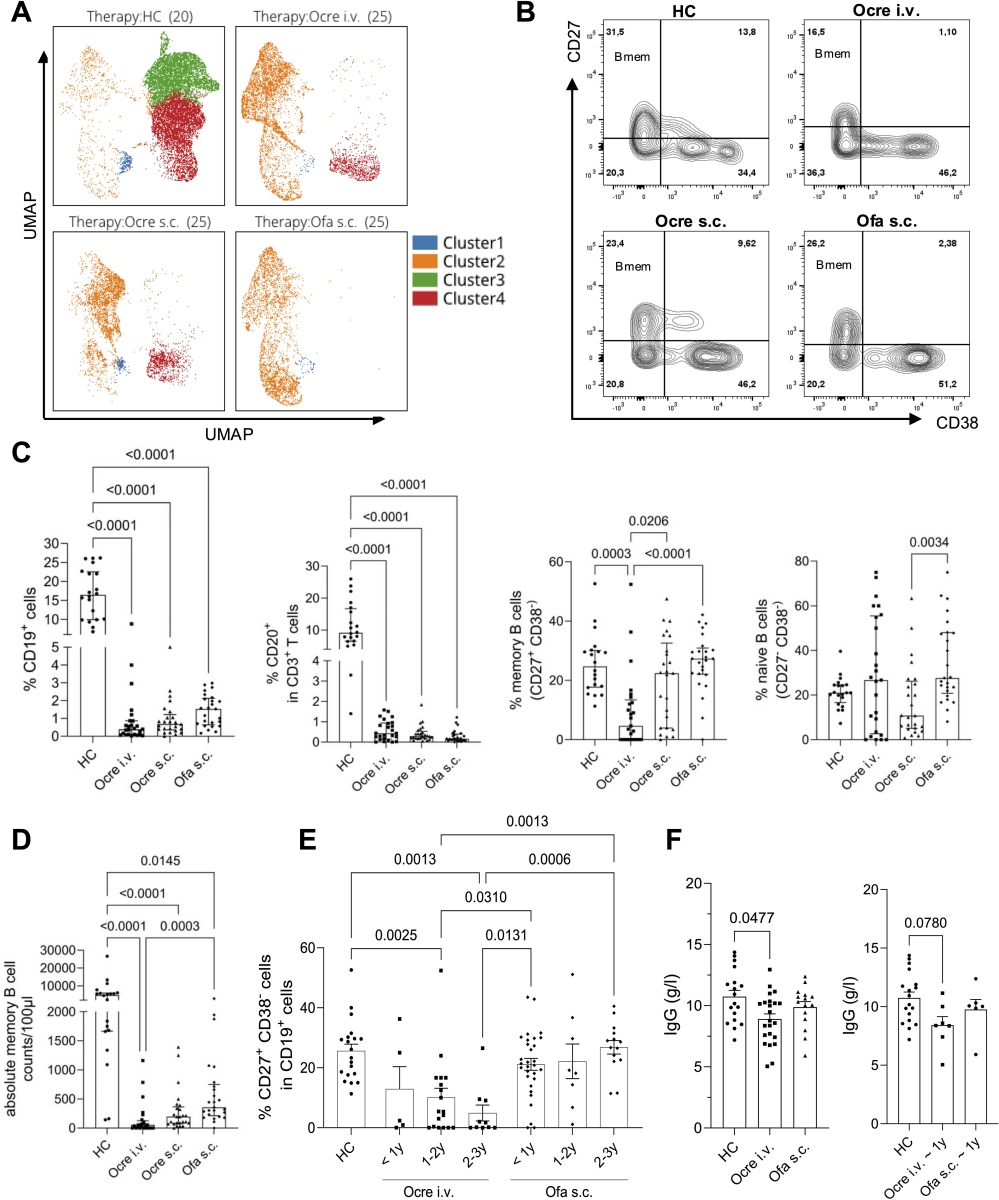
Flow cytometric analysis of B-cell subsets in peripheral blood of Ocre- and Ofa-treated pwMS. **(A)** UMAP plot of CD19^+^ B cells of HC compared to pwMS treated with Ocre i.v., Ocre s.c., and Ofa s.c. confirms distinct effects after B-cell depletion. **(B)** Representative density plot of Bmem percentages in HC compared to Ocre (i.v. and s.c.)- and Ofa-treated pwMS. **(C)** Flow cytometry analysis of CD19^+^ B cell, CD20 expression in CD3^+^ T cell, memory B cell (Bmem; CD27^+^CD38^−^), and naïve B cell (CD27^−^CD38^−^) percentages in peripheral blood of pwMS treated with Ocre (i.v. and s.c.) and Ofa (s.c.) compared to HC. **(D)** Flow cytometry analysis of absolute memory B-cell (CD27^+^CD38^−^) counts in peripheral blood of pwMS treated with Ocre (i.v. and s.c.) or Ofa (s.c.) compared to HC. **(E)** Flow cytometry analysis of Bmem in the blood of HC and subgroups of pwMS with similar therapy durations (<1 year, 1–2 years, and 2–3 years) confirmed a relative increase of Bmem after s.c. B-cell depletion with Ofa. **(F)** IgG levels were analyzed in serum from HC and pwMS treated with i.v. Ocre versus Ofa. Only i.v. Ocre treatment led to a significant reduction of serum IgG levels in pwMS compared to HC. Data were analyzed by Kruskal–Wallis test and Dunn’s multiple comparisons test. Adjusted *p*-values are shown.

We analyzed peripheral blood samples to identify potential immunological differences due to the different B cell-depleting therapies. Age- and gender-matched HC served as a reference for baseline immune cell percentages. UMAP analysis based on flow cytometry data of CD19^+^ B cells (markers: CD19, CD20, CD27, and CD38) revealed distinct clusters between HC and B cell-depleted pwMS (**Figure 1A**). Quantitative analysis of flow cytometry data displayed significantly lower total CD19^+^ B cell percentages in peripheral blood of all Ocre- and Ofa-treated pwMS compared to HC (**Figure 1C**). Relative percentages of CD20-expressing T cells were also significantly lower following B-cell depletion. More in-depth characterization of B-cell subsets showed a lower memory B-cell (Bmem; CD27^+^CD38^−^) percentage in i.v. Ocre-treated pwMS relative to HC. In contrast, B cell-depleted pwMS treated with s.c. Ocre or Ofa showed significantly higher percentages of Bmem compared to Ocre i.v. treated pwMS (**Figures 1B, C**). Quantification of absolute Bmem numbers via True count tubes revealed similar effects of different anti-CD20 therapies (**Figure 1D**). Naïve B cells (CD27^−^CD38^−^) occurred significantly more frequently in pwMS receiving s.c. Ofa compared to those receiving s.c. Ocre B-cell depletion (**Figure 1C**).

We further investigated whether the duration of therapy has a potential influence on the immunomodulatory effects. For this, we compared further blood samples from Ocre- and Ofa-treated pwMS with different therapy durations (<1 year, 1–2 years, and 2–3 years; **Table 2**). Bmem percentages were consistently higher in pwMS treated with s.c. Ofa B-cell depletion compared to i.v. Ocre-treated pwMS (**Figure 1E**).

**Table 2:** Characteristics of B cell-depleted pwMS with similar therapy durations.

Subgroup analysis	Ocre			Ofa		
	<1 year	1–2 years	2–3 years	<1 year	1–2 years	2–3 years
Study participants [*n*]	5	18	10	31	8	14
Age [median years (IQR)]	48 (22–66)	41 (35–52)	49 (32–56.6)	39 (30–51)	49.5 (31.5–52.5)	44 (36.5–52)
EDSS [median (IQR)]	4 (2–4.5)	3.5 (2.1–4.4)	4.3 (2–6)	2.5 (2–3)	2.3 (2–4)	2.5 (1.5–3)
Disease duration [median years (IQR)]	17.9 (13.3–26)	9 (2.8–17)	6.8 (3.9–14.8)	1.8 (0.8–5)	6 (2.4–17.3)	5.7 (3.3–10.5)
Therapy duration [median years (IQR)]	0.7 (0.5–0.8)	1.3 (1–1.5)	2.6 (2.5–2.9)	0.33 (0.08–0.6)	1.2 (1.1–1.5)	2.4 (2–2.7)

Moreover, we assessed serum IgG levels in pwMS who received either i.v. Ocre or s.c. Ofa treatment (**Figure 1F**). Overall, pwMS receiving i.v. Ocre exhibited significantly lower IgG concentrations compared to HC. In contrast, no significant reduction in IgG levels was observed in pwMS treated with s.c. Ofa. Stratification by treatment duration (approximately 1 year of therapy) yielded a consistent pattern: i.v. Ocre treatment was associated with lower IgG levels, whereas Ofa-treated individuals maintained IgG concentrations comparable to those of HC.

### 3.3 Relative increase of memory B cells after switching from intravenous Ocre to subcutaneous Ofa B-cell depletion therapy

**Figure 2:**
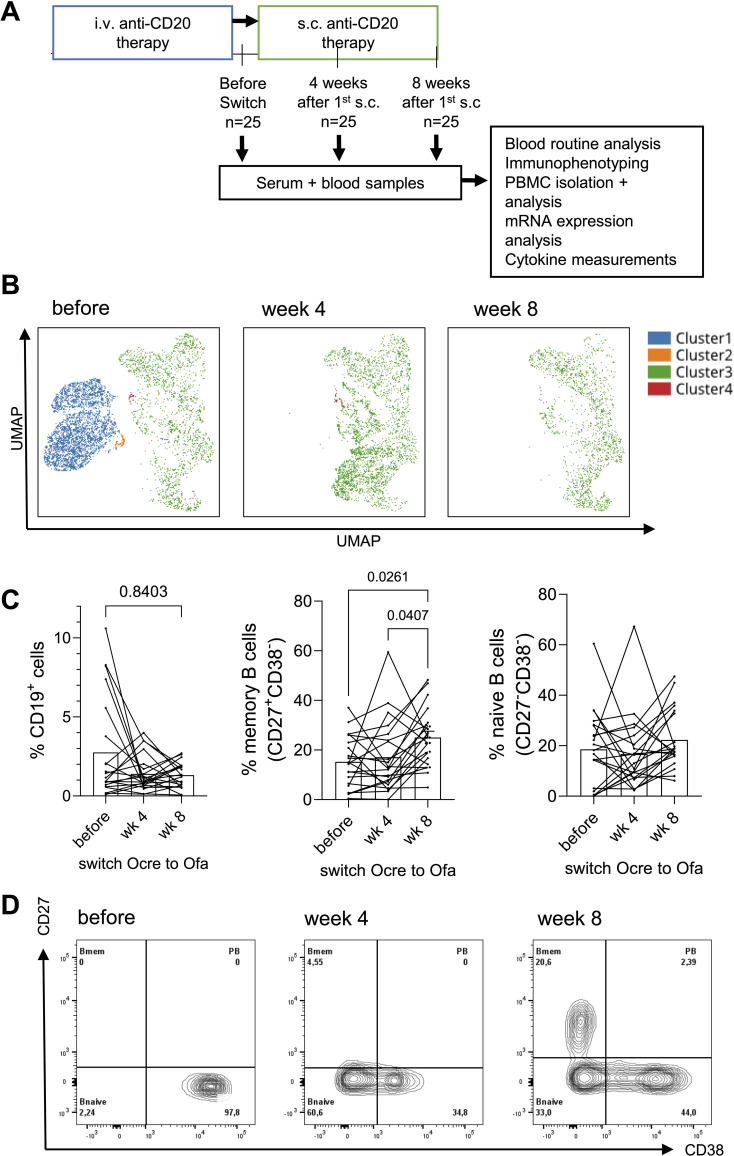
Immunomodulatory effects on B-cell subsets after switching from i.v. to s.c. B-cell depletion. **(A)** Study design to investigate immunomodulatory effects in pwMS after switching from i.v. to s.c. B-cell depletion at three time points: (1) prior to the switch, (2) 4 weeks after the switch, and (3) 8 weeks after the switch. **(B)** UMAP plot of CD19^+^ B cells at the three time points reveals one cluster that disappears after 4 and 8 weeks. **(C)** Flow cytometry analyses of CD19 expressing B-cell percentages, Bmem (CD27^+^CD38^−^) percentages, and naïve B cell (CD27^−^CD38^−^) percentages in pwMS who switched from i.v. to s.c. anti-CD20 therapy. **(D)** Representative density plots showing the expression of CD27 and CD38 in CD19^+^ B cells before the switch and after 4 and 8 weeks. Data were analyzed by Friedman test and Freidman’s multiple comparisons test. Adjusted *p*-values are shown.

To further investigate changes in the B-cell compartment after i.v. Ocre or s.c. Ofa B-cell depletion, a short-term longitudinal analysis was conducted, analyzing *n* = 25 individuals that switched from i.v. Ocre to s.c. Ofa B-cell depletion. In this longitudinal analysis, immune cell compositions were characterized in peripheral blood samples collected at three time points: (1) prior to therapy switch, (2) 4 weeks after the first injection, and (3) 8 weeks after the first injection of Ofa (**Figure 2A**). Flow cytometry analyses of whole blood assessed changes across immune cell populations. Overall, transitioning from i.v. Ocre to s.c. Ofa B-cell depletion did not produce detectable changes in most non-B-cell immune cell subsets (**Supplementary Figure 2**).

UMAP-based analysis of B-cell subsets in CD19^+^ B cells revealed an effect in the B-cell compartment after 4 and 8 weeks following treatment switch (**Figure 2B**). Flow cytometry data analysis showed an expected reduction in CD19^+^ B cells as early as 4 weeks after the first Ofa injection, with a further slight reduction at 8 weeks (**Figure 2C**). B-cell subset analyses indicated a relative increase in Bmem at 8 weeks post-switch (**Figures 2C, D**). Naïve B cells were higher 8 weeks after initiation of s.c. Ofa therapy (**Figure 2C**).

To further explore potential mechanisms underlying these observations, we performed mRNA analyses and serum cytokine concentrations in the switchers. However, neither mRNA expression analyses of B-cell-specific factors nor cytokine measurement revealed conclusive differences between time points (**Supplementary Figures 3** and **4**).

## 4 Discussion

mAbs that target CD20-expressing B cells constitute effective therapies for pwMS. While i.v. administration of Ocre requires ambulatory treatment every 6 months, Ofa represents the first self-administered B cell-depleting drug applied s.c. every 4 weeks (12). Since 2024, Ocre can also be administered s.c., however still ambulatory twice a year. The injection takes approximately 10 minutes, providing a faster alternative to i.v. application (13). Yet, little is known on the differences between different anti-CD20 mAbs on the peripheral immune cell composition.

We here performed cross-sectional in-depth immunophenotyping of peripheral blood immune cell subpopulations of pwMS who received either i.v. Ocre or s.c. Ocre or Ofa B-cell depletion As expected, all treatments efficiently decreased not only B-cell percentages but also CD20-expressing T cells in the peripheral blood of pwMS. Notably, neither population was fully depleted, and a small proportion of residual cells remained detectable in the circulation and was further analyzed. We did not observe any significant changes on other immune cells, including T follicular helper cells, monocytes, natural killer cells, T helper cells, regulatory T cells, or different subpopulations of CD4- or CD8-expressing T cells. Most interestingly, flow cytometry analyses revealed a relative increase of Bmem after s.c. Ocre and Ofa B-cell depletion: pwMS treated with Ofa or Ocre s.c. displayed higher percentages of Bmem in the remaining B cells as compared to i.v. Ocre-treated pwMS and HC. This effect was evident when comparing patients with similar treatment durations and even more pronounced after prolonged therapy duration (2–3 years). We additionally assessed absolute Bmem counts and observed the same pattern, with higher Bmem counts in s.c. Ofa-treated pwMS compared to i.v. Ocre-treated pwMS.

Prior studies that phenotyped immune cell populations in the peripheral blood of pwMS receiving B-cell depletion via i.v. Ocre or s.c. Ofa observed alterations in populations other than the B-cell compartment (14–16). One study observed a shift towards a transitional and activated, IgA^+^ switched memory B cell type in the residual B-cell pool after i.v. Ocre treatment (16). Moreover, longitudinal analyses in pwMS treated with either Ocre or Ofa revealed a general depletion of all B-cell subsets after 12 months of therapy (15). In contrast, our study investigated both a cross-sectional analysis of pwMS with longer therapy durations (2–3 years) and a short-term longitudinal analysis of early immunological effects following a switch from i.v. Ocre to s.c. Ofa therapy. Here, an increase in Bmem percentages could be observed relatively early after the therapy switch from i.v. Ocre to s.c. Ofa treatment, occurring as soon as 4 weeks after the first s.c. Ofa injection. This effect persisted until week 8 after the switch. Yet, potential long-term effects need to be investigated in additional analyses at later time points after a therapy switch. Moreover, these short-term longitudinal data do not provide evidence for causal effects and must be interpreted as exploratory. A complete understanding on the relation of immunological changes occurring in the longitudinal 8-week observational period to the cross-sectional assessments after years of treatment needs further analyses. The clinical consequences of the observed Bmem increase in pwMS remain unclear to date. Yet, previous work has shown that a lateral switch between Ocre and Ofa treatment does not affect treatment effectiveness outcomes (17). Comparisons of the safety profiles and disease control in pwMS initiating Ocre and Ofa therapy resulted in similar outcomes (18).

Whether the observed effect of increasing Bmem is associated with differences in the therapeutic antibodies or the route of administration remains speculative. Ocre and Ofa target distinct epitopes on CD20 that might influence the B-cell depletion (8, 10). The two antibodies also differ in their pharmacokinetic profiles, particularly in the rate at which they reach systemic circulation (19). S.c. administered Ofa enters the systemic circulation via an indirect route through the lymphatic system, resulting in a distinct tissue distribution, particularly within lymph nodes, compared to an i.v. administration (20). This has also been demonstrated in murine models of experimental autoimmune encephalomyelitis (21, 22). The findings of our exploratory study provide a first hint that there might be a difference in the route of administration of distinct anti-CD20 mAbs. This could be a potential cornerstone for further mechanistic studies. Yet, the analyzed cohorts include only a limited number of patients (*n* = 25 each). Therefore, the effect remains to be elucidated in larger cohorts.

A further study limitation might be the potential impact of prior disease-modifying therapies. However, this effect is likely minimal in our cohort, as patients in the Ocre i.v. group had received B-cell depletion for a median duration of 2.4 years, and the s.c. groups mainly comprised patients previously treated with i.v. B-cell-depleting therapy. Therefore, any effects of prior therapies (other than B-cell depletion) are expected to have attenuated over time. Moreover, as a single-center study with patients recruited from one hospital, our findings cannot be generalized to the broader pwMS population. Larger cohorts and longitudinal studies are warranted to validate our results. Although we observed changes in mRNA expression and cytokine profiles, these findings currently do not provide causal evidence for a potential underlying mechanism. Such interpretations remain speculative and need to be confirmed in future studies. A further limitation is the use of subgroup analyses, for which a risk of type I error remains. All reported *p*-values were adjusted; however, the analyses are regarded as exploratory and hypothesis-generating rather than confirmatory in nature. In addition, residual confounding of age, sex, EDSS score, therapy, or treatment duration remains possible, and the observed associations should be interpreted with caution. Therefore, adequately powered prospective studies using multivariable modeling approaches will be necessary to clarify the relative contribution of treatment-related and clinical variables.

Serum IgG levels from B cell-depleted pwMS were analyzed and revealed lower IgG concentrations in i.v. Ocre-depleted pwMS compared to HC, whereas s.c. Ofa-treated pwMS maintained IgG concentrations comparable to HC. However, the clinical significance of this finding was not investigated within this study, but might be comparable with already published data (23–26).

The clinical implications of increased Bmem percentages remain unclear, yet our study adds evidence to the immunological differences between different B-cell depletion therapies in MS. Further studies are needed to elucidate how different antibodies and administration routes might influence immunological outcomes.

## Data Availability

The original contributions presented in the study are included in the article/**Supplementary Material**. Further inquiries can be directed to the corresponding author.
