# Supplementary material for: Relative increase of memory B-cell subsets under s.c. B-cell-depleting therapies in multiple sclerosis
Source: Front Immunol. 2026 Jun 15;17:1812392. doi: 10.3389/fimmu.2026.1812392 (PMC13310750; doi:10.3389/fimmu.2026.1812392)
Supplement: Supplementary file 1 [file DataSheet1.docx]

Supplementary Material

# Supplementary Methods

## Cytokine measurements

To detect cytokines in serum samples from pwMS, enzyme-linked immunosorbent assays (ELISA) were performed for APRIL, BAFF (R&D Systems), IL-9, IL-10, IL-6 and GM-CSF (BioLegend) according to the manufacturer’s instructions.

## Real-time polymerase chain reaction (qPCR)

Gene expression analyses were performed via quantitative real-time polymerase chain reaction. RNA of PBMCs was isolated using the RNase Mini Kit #A2791 (Qiagen). RNA yield was quantified by absorbance measurements at 260 nm. cDNA synthesis was conducted with the GoScript Reverse Transcription Mix (Promega). For qPCR, the Kapa Probe Fast qPCR Master Mix (SigmaAldrich) was used and reactions were performed in triplicates on the qTower3G Real-Time Thermocycler (Analytik Jena). GAPDH was used as a housekeeper gene to normalize values. The relative quantification was analysed using the delta-delta Ct method.

## Flow cytometry

Compensation was performed using the *BD FACSCanto II Flow Cytometer* with the help of single-stain controls that were measured and the compensation matrix was then calculated by the *BD FACSDiva Software*. To avoid batch effects, we consistently used standardized protocols, the same voltage settings during measurements and employed *CS&T Beads* from BD. The gating strategy is shown in Supplementary Figure 1. The gates were set based on the first sample measurement, and applied to all samples using the same parameters.

# Supplementary Figures

**
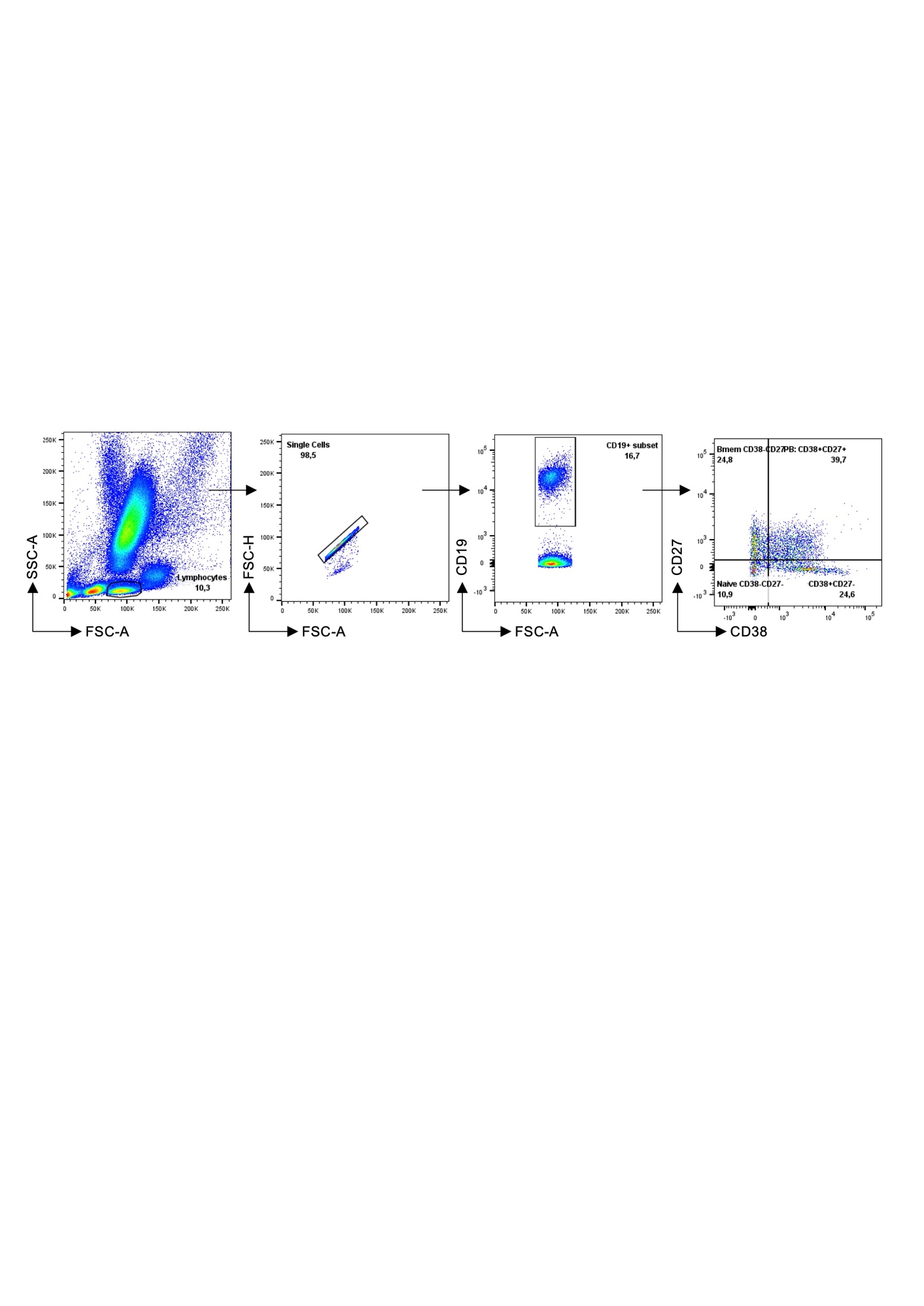
Supplementary Figure 1.** Flow cytometry gating strategy for B cell phenotyping in whole blood.

**
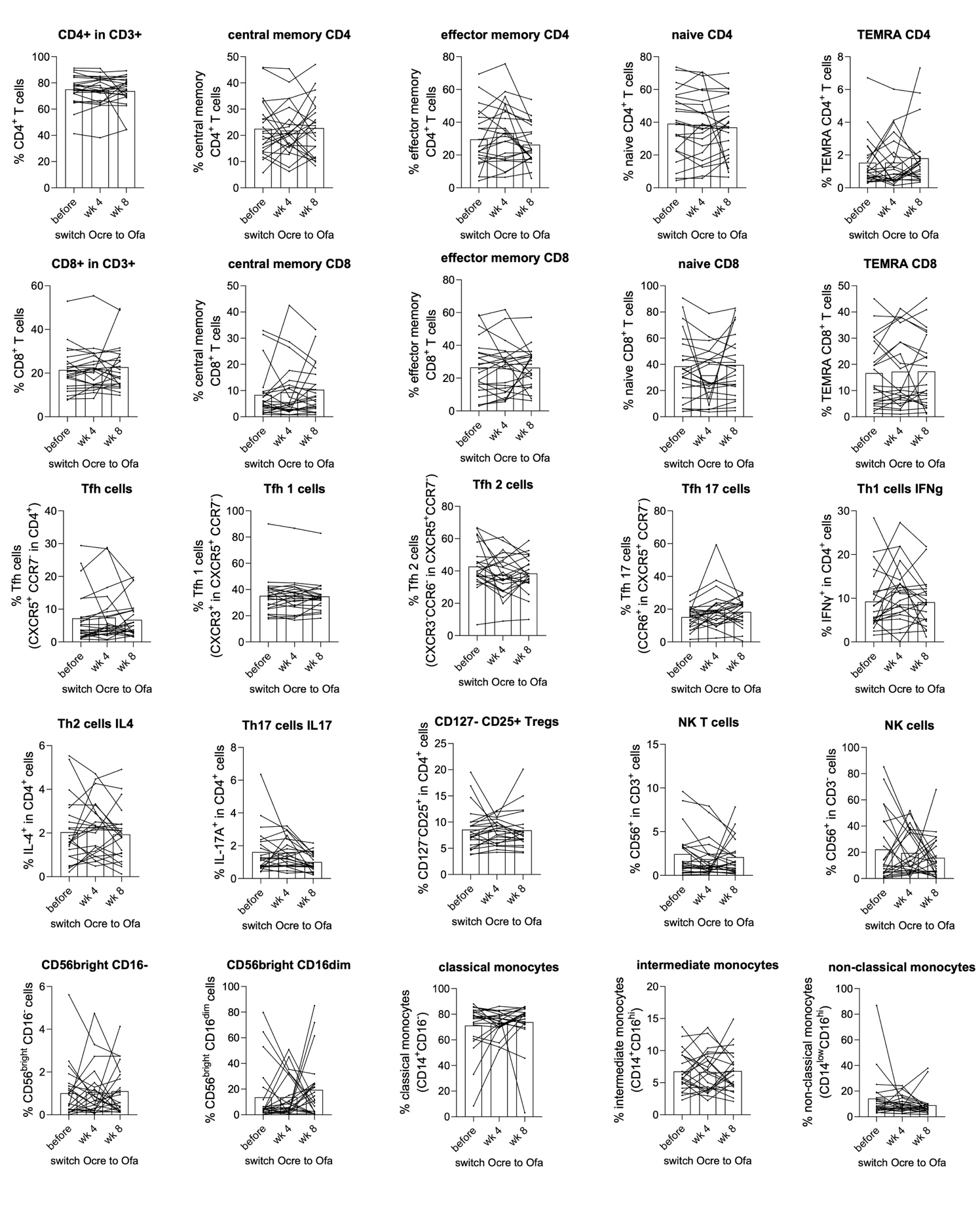
**

**Supplementary Figure 2.** Flow cytometry analysis of several immune cell populations in pwMS that switched from i.v. to s.c. B cell depletion.

**
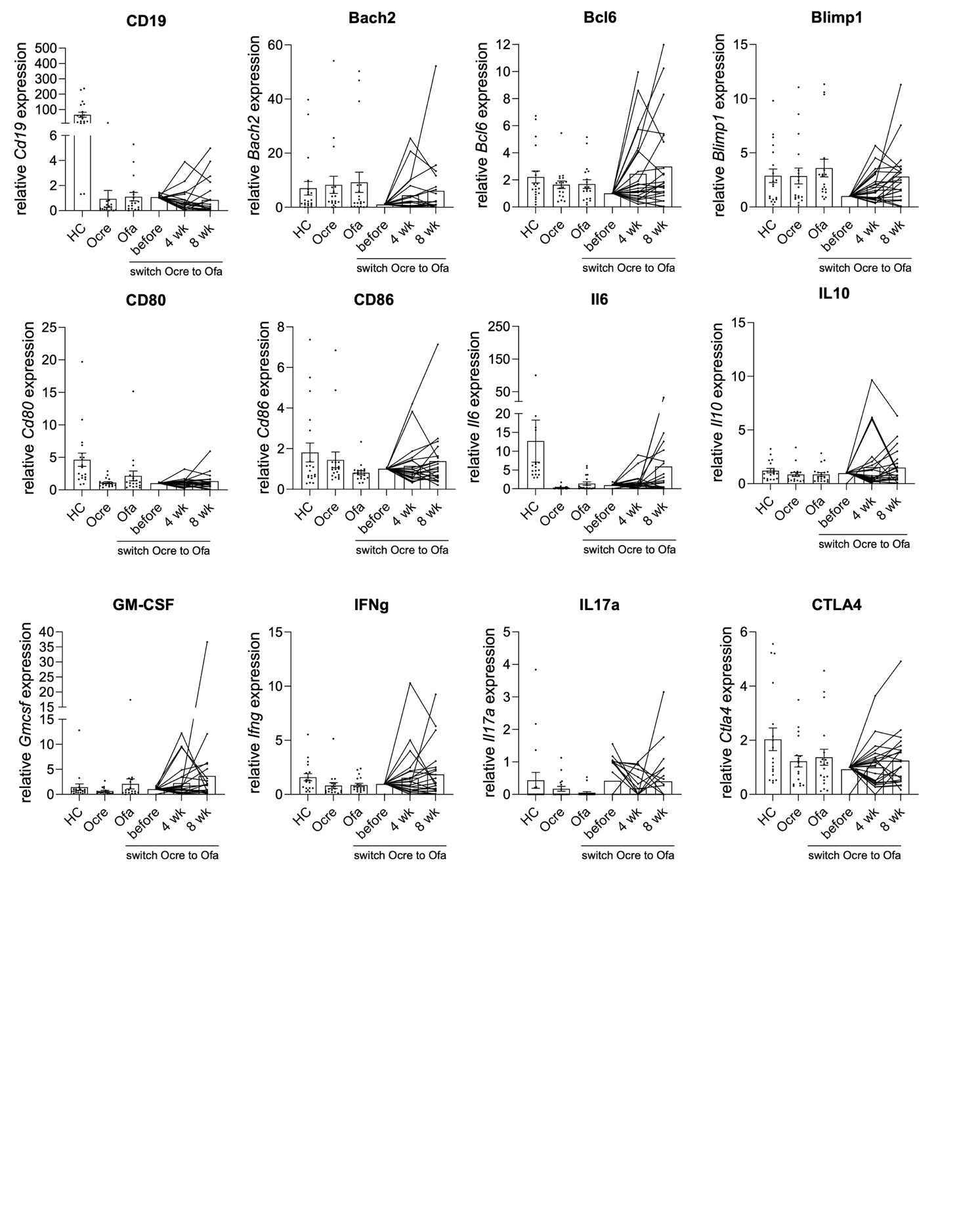
**

**Supplementary Figure 3.** mRNA expression analysis of pwMS that switched from i.v. to s.c. anti-CD20 therapy.

**
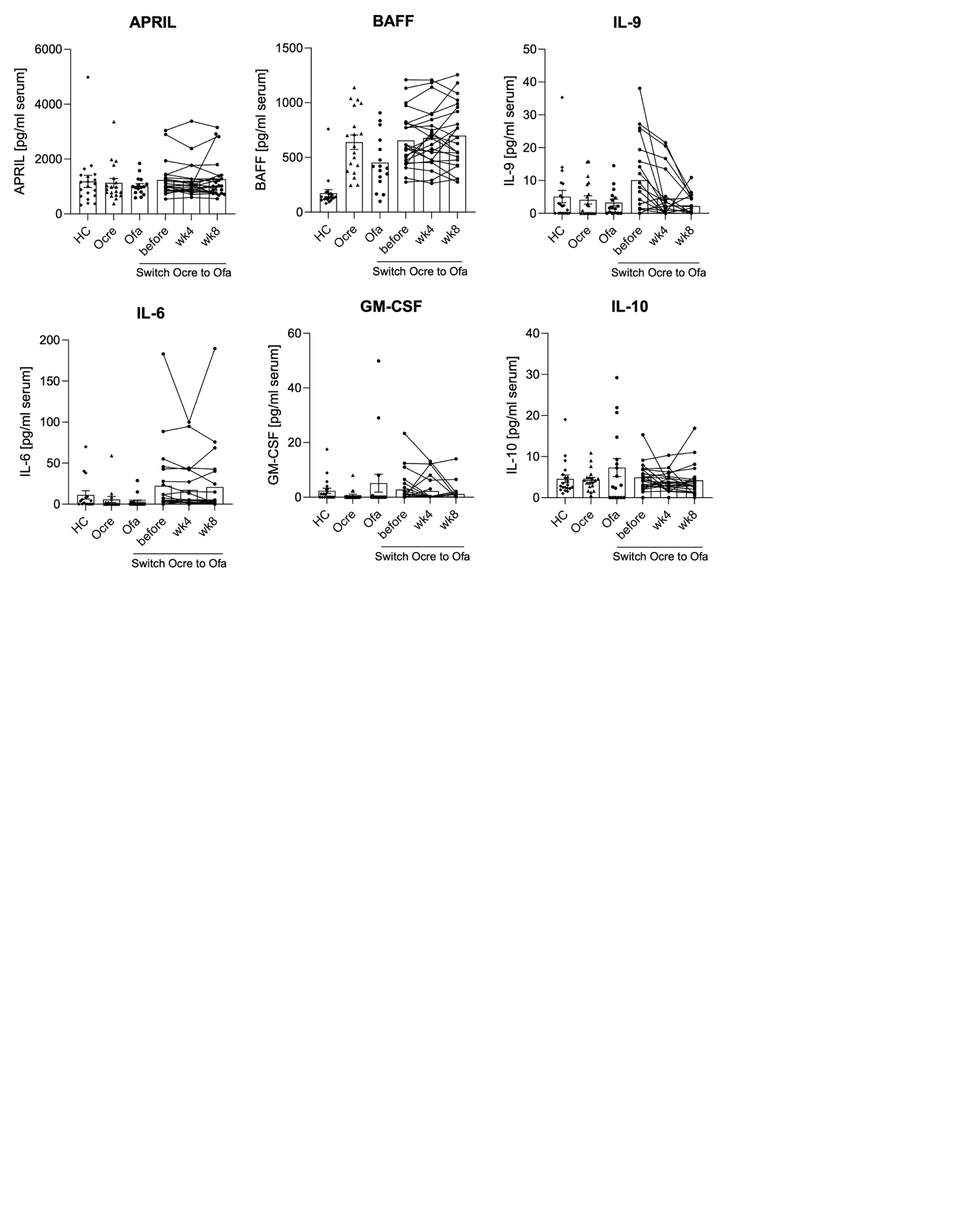
**

**Supplementary Figure 4.** Cytokine secretion in serum of pwMS that switched from i.v. to s.c. B cell depletion therapy.

# Supplementary Tables

**Supplementary table 1. Antibodies and controls used for flow cytometry analysis.**

| **Antigen** | **Conjugate** | **Clone** | **Isotype** | **Vendor** | **RRID** |
| --- | --- | --- | --- | --- | --- |
| CD3 | APC | REA613 | Recombinant human IgG1 | Miltenyi Biotec | AB_2725963 |
| CD4 | FITC | REA623 | Recombinant human IgG1 | Miltenyi Biotec | AB_2726690 |
| CD8 | VioBlue | REA734 | Recombinant human IgG1 | Miltenyi Biotec | AB_2659239 |
| CD14 | FITC | REA599 | Recombinant human IgG1 | Miltenyi Biotec | AB_2655049 |
| CD16 | VioBlue | REA423 | Recombinant human IgG1 | Miltenyi Biotec | AB_2726154 |
| CD19 | PE-Vio770 | REA67 | Recombinant human IgG1 | Miltenyi Biotec | AB_2726200 |
| CD20 | PE | REA780 | Recombinant human IgG1 | Miltenyi Biotec | AB_2656064 |
| CD25 | APC | REA570 | Recombinant human IgG1 | Miltenyi Biotec | AB_2733791 |
| CD27 | VioBright515 | REA499 | Recombinant human IgG1 | Miltenyi Biotec | AB_2751971 |
| CD38 | VioBright423 | REA572 | Recombinant human IgG1 | Miltenyi Biotec | AB_2904977 |
| CD45RO | PE-Vio770 | REA611 | Recombinant human IgG1 | Miltenyi Biotec | AB_2751129 |
| CD56 | PE | REA196 | Recombinant human IgG1 | Miltenyi Biotec | AB_2726090 |
| CD127 | PE-Vio770 | REA614 | Recombinant human IgG1 | Miltenyi Biotec | AB_2733198 |
| CCR6 | VioBright423 | REA190 | Recombinant human IgG1 | Miltenyi Biotec | AB_2904798 |
| CCR7 | PE | REA546 | Recombinant human IgG1 | Miltenyi Biotec | AB_2751741 |
| CXCR3 | PE | REA232 | Recombinant human IgG1 | Miltenyi Biotec | AB_2752105 |
| CXCR5 | PE-Vio770 | REA103 | Recombinant human IgG1 | Miltenyi Biotec | AB_2733205 |
| FOXP3 | PE | REA1253 | Recombinant human IgG1 | Miltenyi Biotec | AB_2889668 |
| IL4 | PE | REA895 | Recombinant human IgG1 | Miltenyi Biotec | AB_2726798 |
| IL17A | PE-Vio770 | REA1063 | Recombinant human IgG1 | Miltenyi Biotec | AB_2751469 |
| IFNg | APC | REA600 | Recombinant human IgG1 | Miltenyi Biotec | AB_2751118 |
| REA control | PE | REA293 | Recombinant human IgG1 | Miltenyi Biotec | AB_2857433 |
| REA control | PE-Vio770 | REA293 | Recombinant human IgG1 | Miltenyi Biotec | AB_2921861 |
| REA control | APC | REA293 | Recombinant human IgG1 | Miltenyi Biotec | AB_2784399 |
| REA control | VioBright423 | REA293 | Recombinant human IgG1 | Miltenyi Biotec | AB_2905377 |
| REA control | FITC | REA293 | Recombinant human IgG1 | Miltenyi Biotec | AB_2751490 |
